# Reactive Oxygen Species in the Adverse Outcome Pathway Framework: Toward Creation of Harmonized Consensus Key Events

**DOI:** 10.3389/ftox.2022.887135

**Published:** 2022-07-06

**Authors:** Shihori Tanabe, Jason O’Brien, Knut Erik Tollefsen, Youngjun Kim, Vinita Chauhan, Carole Yauk, Elizabeth Huliganga, Ruthann A. Rudel, Jennifer E. Kay, Jessica S. Helm, Danielle Beaton, Julija Filipovska, Iva Sovadinova, Natalia Garcia-Reyero, Angela Mally, Sarah Søs Poulsen, Nathalie Delrue, Ellen Fritsche, Karsta Luettich, Cinzia La Rocca, Hasmik Yepiskoposyan, Jördis Klose, Pernille Høgh Danielsen, Maranda Esterhuizen, Nicklas Raun Jacobsen, Ulla Vogel, Timothy W. Gant, Ian Choi, Rex FitzGerald

**Affiliations:** ^1^ Division of Risk Assessment, Center for Biological Safety and Research, National Institute of Health Sciences, Kawasaki, Japan; ^2^ Wildlife Toxicology Research Section, Environment and Climate Change Canada, Toronto, ON, Canada; ^3^ Norwegian Institute for Water Research (NIVA), Oslo, Norway; ^4^ Norwegian University of Life Sciences (NMBU), Ås, Norway; ^5^ Centre for Environmental Radioactivity, Norwegian University of Life Sciences (NMBU), Ås, Norway; ^6^ Korea Institute of Science and Technology (KIST) Europe, Saarbrücken, Germany; ^7^ Health Canada, Ottawa, ON, Canada; ^8^ University of Ottawa, Ottawa, ON, Canada; ^9^ Silent Spring Institute, Newton, MA, United States; ^10^ Canadian Nuclear Laboratories, Chalk River, ON, Canada; ^11^ Independent Researcher, Ohrid, North Macedonia; ^12^ RECETOX, Faculty of Science, Masaryk University, Brno, Czech Republic; ^13^ U.S. Army Engineer Research and Development Center (ERDC), Vicksburg, MS, United States; ^14^ Department of Toxicology, University of Würzburg, Würzburg, Germany; ^15^ National Research Centre for the Working Environment, Copenhagen, Denmark; ^16^ Organisation for Economic Co-operation and Development (OECD), Paris, France; ^17^ Group of Alternative Method Development for Environmental Toxicity Testing, IUF—Leibniz-Research Institute for Environmental Medicine, Duesseldorf, Germany; ^18^ Philip Morris International R&D, Philip Morris Products SA, Neuchatel, Switzerland; ^19^ Center for Gender-specific Medicine, Italian National Institute of Health, Rome, Italy; ^20^ University of Helsinki, Ecosystems and Environment Research Programme, Faculty of Biological and Environmental Sciences, Lahti, Finland, and Helsinki Institute of Sustainability Science (HELSUS), Helsinki, Finland; ^21^ UK Health Security Agency, Public Health England, London, United Kingdom; ^22^ Universities of Basel and Geneva, Basel, Switzerland

**Keywords:** adverse outcome pathway (AOP), oxidative stress, reactive nitrogen species (RNS), disease, reactive oxygen species (ROS)

## Abstract

Reactive oxygen species (ROS) and reactive nitrogen species (RNS) are formed as a result of natural cellular processes, intracellular signaling, or as adverse responses associated with diseases or exposure to oxidizing chemical and non-chemical stressors. The action of ROS and RNS, collectively referred to as reactive oxygen and nitrogen species (RONS), has recently become highly relevant in a number of adverse outcome pathways (AOPs) that capture, organize, evaluate and portray causal relationships pertinent to adversity or disease progression. RONS can potentially act as a key event (KE) in the cascade of responses leading to an adverse outcome (AO) within such AOPs, but are also known to modulate responses of events along the AOP continuum without being an AOP event itself. A substantial discussion has therefore been undertaken in a series of workshops named “Mystery or ROS” to elucidate the role of RONS in disease and adverse effects associated with exposure to stressors such as nanoparticles, chemical, and ionizing and non-ionizing radiation. This review introduces the background for RONS production, reflects on the direct and indirect effects of RONS, addresses the diversity of terminology used in different fields of research, and provides guidance for developing a harmonized approach for defining a common event terminology within the AOP developer community.

## 1 Introduction

Exposure to various types of stressors (e.g., allergens, ionizing radiation, chemicals) can induce cellular-level oxidative damage to macromolecules such as DNA, proteins, and lipids. This is a result of oxidative stress from an imbalance in the production of reactive oxygen species (ROS) in a wide sense. One of the difficulties in the field of ROS research is the use of terminology whose meaning is interpreted differently in the different areas of expertise. To overcome the problems, the Mystery of ROS, an international consortium for creating harmonized Key Events (KEs) on ROS in the Adverse Outcome Pathway (AOP) framework has been established (Tanabe et al., 2022). This review aims to provide some guidance on the definition of the harmonized KEs on ROS in the AOP framework.

The field of ROS in a broad sense has previously been reviewed by Sies et al. (Sies et al., 2017). Non-radical species function as second messengers to regulate life (Sies et al., 2017). Two main types of radicals can be produced including ROS in a narrow sense and reactive nitrogen species (RNS), which are regulated by antioxidant defense response (ADR) that might be defined as radical scavenging mechanisms and oxidant reduction. The ADR constitutes the totality of the activation of processes that protect the cells against ROS. These radicals can be long- and short-lived, depending on their reactivity with other molecules. In order to handle ROS (in a narrow sense) and RNS formation, antioxidant defense mechanisms are recruited to manage the damage. These processes can be classified as enzymatic [e.g., glutathione-*S*-transferase (GST), catalase (CAT), glutathione-peroxidase (GPX), and superoxide dismutase (SOD)] and non-enzymatic systems (e.g., uric acid, vitamin C and E and lipoic acid). Normally, these are the first line of defense and these can act as oxidant scavengers. Oxidative stress is defined as a condition where the ROS production is sustainably excessive beyond ADRs and plays a central role in pathological processes including cancer, diabetes, chronic kidney disease, neurodegenerative disease, cardiovascular disease, chronic obstructive pulmonary disease (Dröge, 2002; Liguori et al., 2018). ROS are also crucial in generating immune responses, signaling injury, and upregulating inflammatory responses, thereby strongly affecting various disease pathogenesis, including cancer and bacterial and viral infection including severe acute respiratory syndrome coronavirus-2 (SARS-CoV-2) causing the current global pandemic coronavirus infectious disease (COVID-19) (Liou and Storz, 2010; Cecchini and Cecchini, 2020; Saleh et al., 2020; Shenoy, 2020; Alam and Czajkowsky, 2021; Kalyanaraman, 2021). ROS and oxidative stress have been implicated as potential key contributors to toxic effects mediated by pollutants, radiation, and nanoparticles. Environmental stressors such as pollutants and radiation often contribute to the exaggeration of oxidative stress (Table 1).

**TABLE 1:** Stressors and diseases related to reactive oxygen species and oxidative stress.

Stressors	Diseases/Toxic Effects
Autoimmune disorders and allergens	Tissue damage Valko et al. (2007); Di Florio et al. (2020); Seebacher et al. (2021), Respiratory disease Racanelli et al. (2018)
Chemotherapy and xenobiotics	Cell death Conklin. (2004), Tumor formation Henkler et al. (2010); Seebacher et al. (2021)
Tobacco and alcohol	Pulmonary disease Chen et al. (2019); Seebacher et al. (2021), Breast cancer Wang et al. (2017)
Ionizing and non-ionizing radiation	Multiple cancers, including breast cancer, gastric cancer and liver cancer Sauvaget et al. (2005); Bhattacharyya et al. (2014); Yusefi et al. (2018); Helm and Rudel. (2020); Kuo et al. (2021), Erythema Clydesdale et al. (2001); Seebacher et al. (2021)
Bacterial and viral infection	Organ damage and malignancy Ivanov et al. (2017); Seebacher et al. (2021), Liver disease Đorđević et al. (2021)
Severe acute respiratory syndrome coronavirus- 2 (SARS-CoV-2)	Hyperinflammation/cytokine storm Frisoni et al. (2021); Kaidashev et al. (2021), Thrombosis and disseminated intravascular coagulation Aid et al. (2020); Connors and Levy. (2020); Mackman et al. (2020)
Nanoparticles	Lung injury, including inflammation, fibrosis and cancer Huh et al. (2010); Lu et al. (2014); Halappanavar et al. (2020); Nymark et al. (2021), Liver toxicity Yao et al. (2019)

## 2 Intracellular Sources of ROS and Initiating Events

ROS can be both KEs in the AOP leading to the Adverse Outcome (AO) or associated where their formation is a consequence of the AO rather than a cause of that AO. For all environmental stressors, it is crucial to make this distinction. This can be achieved by determining the temporal relationship of ROS formation relative to the AO or examining the Molecular Initiating Event (MIE). Where for example, the MIE is likely to generate ROS such as a redox cycling or effect on complex I there is a higher probability of ROS being a KE in the AOP than where the MIE is something unrelated to ROS such as direct reaction with protein or DNA.

### 2.1 Direct Sources of ROS

Radiation can increase immediate and longer-term ROS. Comprehensive mechanisms of ROS-dependent and independent DNA damage induced by ionizing radiation and solar radiation have been proposed (Cadet and Wagner, 2013; Helm and Rudel, 2020). Radiation can also increase antioxidants such as SOD, CAT, and peroxidase (POD) (Seen and Tong, 2018; Ahmad et al., 2021). Radiation-induced DNA damage and ROS can also lead to cell death and autophagy and are utilized for this reason in cancer treatment (Ma et al., 2021; Yamamoto et al., 2021). The differences between high and low linear energy transfer (LET) should be considered in terms of ROS production and DNA damage in the radiation fields of cancer since high LET deposits dense clusters of energy closer to the tissue surface and creates more complex DNA damage (Goodhead, 1988).

### 2.2 Indirect Sources of ROS

ROS are commonly involved in the mode of action (MoA) of various classes of environmental chemicals including metals such as lead, chromium, arsenic, mercury, nickel, and cadmium (Renu et al., 2021), pesticides such as pyrethroids, carbamates and organophosphates (Medithi et al., 2021), and other industrial chemicals such as bisphenol A (Steffensen et al., 2020). ROS production contributes to a large variety of environmentally-induced diseases (Omidifar et al., 2021), e.g., oxidative stress is one of the most important mechanisms of action of pesticides in acute and chronic poisoning (Lukaszewicz-Hussain, 2010), and of metals in hepatotoxicity (Renu et al., 2021). Mitochondria are one of the main producers of ROS in eukaryotic cells. The sources of ROS include mitochondria in muscle cells, nicotinamide adenine dinucleotide phosphate (NADPH) oxidase (NOX), phospholipase A_2_ (PLA_2_), xanthine oxidase, monoamine oxidase (MAO), dehydrogenase, and immune cells such as macrophages, eosinophils, monocytes or neutrophils (Powers and Jackson, 2008; Vargas-Mendoza et al., 2021). Free adenosine diphosphate (ADP), inorganic phosphate (P_i_), and O_2_ activate the electron transport chain (ETC) in mitochondria (Hargreaves and Spriet, 2020; Vargas-Mendoza et al., 2021). Other sources are also important as ROS producers. Angiotensin II (Ang II), the main product and effector of the renin-angiotensin system stimulation, is a regulator of blood pressure and produces ROS by stimulating NOX (Nguyen Dinh Cat et al., 2013; Forrester et al., 2018; Pothen and Balligand, 2021). Ang II-induced ROS production leads to mitogen-activated protein kinase (MAPK) activation, where ROS play a role as a second messenger (Nishida et al., 2005; Forrester et al., 2018). When excess Ang II binds to the Ang II Type 1 Receptor (AT_1_R), several signaling pathway cascades, including the ROS signaling. The formation of ROS through NOX activation further regulates AT_1_R through a feed-forward mechanism, which amplifies the ROS production with a feed-forward mechanism (Datla and Griendling, 2010; Frazziano et al., 2014). There are varying NOX isoforms that could possibly be engaged in this mechanism which includes NOX 4 isoform located in mitochondria (Veith et al., 2019; Fukai and Ushio-Fukai, 2020). The NOX-derived ROS stimulates peptides and cell-surface receptors to initiate intracellular signaling pathways. Both the ROS formulation and Ang II/AT_1_R stimulations regulate MAPK pathways, modulating the transcription factors. Multiple MAPK-mediated transcription factors drive the accumulation of myofibroblasts and the development of lung fibrosis (Zhao et al., 2007; Hardie et al., 2009). In the case of alveolar cells, Ang II accumulates at the cellular level resulting in inflammation and lung fibrosis as an AO (Parimon et al., 2020; Shi et al., 2021). Still, the non-converted Ang II, induced by angiotensin converting enzyme (ACE) II downregulation, could circulate to other parts of the body, such as the live heart, skin, kidneys, blood vessels, skeletal muscles, and brain, causing similar AT_1_R activation results in other organs to induces ROS mediated vasoconstriction, proliferation, inflammation, and fibrosis. In addition, some nanomaterials, especially carbon-based nanomaterials, are efficient ROS producers (Jacobsen et al., 2008).

### 2.3 Methodologies for Measuring Oxidative Stress

ROS can be detected by intracellular ROS assay and *in vitro* ROS/RNS assay. Nitric oxide can be detected in intracellular nitric oxide assay. ROS detection in biological systems includes spectrophotometry methods, fluorescence and chemiluminescence methods, and electron-spin resonance (ESR), which requires probes that produce stable products (Dikalov and Harrison, 2014; Zhang et al., 2018). Adducts produced by covalent binding with free radicals can be detected by ESR (Dikalov and Harrison, 2014). Hydroxyl, peroxyl, or other ROS can be measured using a fluorescence probe, 2′, 7′-dichlorodihydrofluorescein diacetate (DCFH-DA), at fluorescence detection at 480 nm/530 nm. Recent progress in ROS measurement includes the development of a standard operating procedure (SOP) for DCFH-DA acellular assay (Boyles et al., 2022). Chemiluminescence analysis can detect the superoxide, where some probes have a wider range for detecting hydroxyl radical, hydrogen peroxide (H_2_O_2_), and peroxynitrite (Fuloria et al., 2021). ROS in the blood can be detected using superparamagnetic iron oxide nanoparticles (SPION)-based biosensor (Lee et al., 2020). H_2_O_2_ can be detected with a colorimetric probe, which reacts with H_2_O_2_ in a 1:1 stoichiometry to produce a bright pink colored product, followed by the detection with a standard colorimetric microplate reader with a filter in the 540–570 nm range. The levels of ROS can be quantified using multiple-step amperometry using a stainless steel counter electrode and non-leak Ag|AgCl reference node (Flaherty et al., 2017). Singlet oxygen can be measured by monitoring the bleaching of *p*-nitrosodimethylaniline at 440 nm using a spectrophotometer with imidazole as a selective acceptor of singlet oxygen (Onoue et al., 2013).

The level of CAT, GPX, or SOD can be measured as enzymes in the cellular oxidative defense system. CAT is an anti-oxidative enzyme that catalyzes the resolution of H_2_O_2_ into H_2_O and O_2_. The chemiluminescence or fluorescence of HRP catalytic reaction can be detected with residual H_2_O_2_ and probes [DHBS + AAP, or ADHP (10-acetyl-3, 7-dihydroxyphenoxazine)]. Anti-oxidant capacity is also one of the oxidative stress markers. Oxygen radical antioxidant capacity, hydroxyl radical antioxidant capacity, total antioxidant capacity, the cell-based exogenous antioxidant assay can be used for measuring the antioxidant capacity. Oxidation of protein can be measured by the detection of protein carbonyl content (PCC), 3-nitrotyrosine, advanced oxidation protein products, or BPDE protein adduct. DNA oxidation can be detected with 8-oxo-dG/8-hydroxy-2′-deoxyguanosine (8-OHdG) by ELISA. Lipid peroxides decompose to form malondialdehyde (MDA) and 4, hydroxynonenal (4-HNE), natural bi-products of lipid peroxidation. Lipid peroxidation can be monitored by thiobarbituric acid (TBA) reactive substances in biological samples. MDA and TBA form MDA-TBA adduct in a 1:2 stoichiometry and are detected by colorimetric or fluorometric measurement. While direct ROS determination, a promising oxidative stress biomarker, is challenging due to the short half-life and high reactivity of ROS, detection of the resulting oxidative damage to biomolecules (DNA, lipids, and proteins) and antioxidant status (enzymatic antioxidant activities and non-enzymatic antioxidant levels) is more reliable in biological setting (Katerji et al., 2019).

## 3 Relationships Between ROS and Pathogenesis

### 3.1 The Roles of ROS in Diseases

Prolonged ROS and oxidative stress mediate a variety of diseases (e.g., cancer, neurological disorders, cardiac diseases, pulmonary diseases), indicating that ROS and oxidative stress can be causal factors as well as biomarkers of diseases (Annesley and Fisher, 2019; Kay et al., 2019; Climent et al., 2020; Ghezzi, 2020). ROS can be classified as free radicals (Table 2) and non-radical (Table 3) ROS. The term RNS refers to both nitrogen-centered radicals and other reactive molecules, which can induce nitrosative stress (e.g., nitric oxide, nitrogen dioxide, nitrous acid, peroxynitrite, dinitrogen trioxide) (Powers and Jackson, 2008). In general, the timely detection of “live-ROS” is difficult since ROS have an extremely short half-life (t½ in seconds). On the other hand, the level of antioxidant enzymes and products of oxidation can be detected as indicators of redox state and oxidative stress, respectively (Gornicka et al., 2011). For instance, 7, 8-dihydro-8-oxo-2′-deoxyguanosine or 7, 8-dihydro-8-oxoguanosine can be detected as a DNA damage biomarker in cancer, and advanced glycation end products or MDA are biomarkers in diabetes (Liguori et al., 2018). An antioxidant enzyme NAD(P)H quinone dehydrogenase 1 (*NQO1*) is up-regulated while *CAT* is down-regulated in nonalcoholic steatohepatitis livers (Gornicka et al., 2011).

**TABLE 2:** Free radicals.

Name	Molecular formula
Superoxide anion	O_2_^·-^
Hydroxyl radical	·OH
Nitric oxide	·NO
Nitrogen dioxide	·NO_2_
Organic radicals	R·
Peroxyl radicals	ROO·
Alkoxyl radicals	RO·
Thiyl radicals	RS·
Sulfonyl radicals	ROS·
Thiyl peroxyl radicals	RSOO·
Disulfides	RSSR

**TABLE 3:** Non-radical ROS.

Name	Molecular formula
Hydrogen peroxide	H_2_O_2_
Singlet oxygen	^1^O_2_
Ozone/trioxygen	O_3_
Organic hydroperoxides	ROOH
Hypochlorite	ClO^−^
Peroxynitrite	ONOO^−^
Nitrosoperoxycarbonate anion	O=NOOCO_2_^−^
Nitrocarbonate anion	O_2_NOCO_2_^−^
Dinitrogen dioxide	N_2_O_2_
Nitronium	NO_2_^+^
Highly reactive lipid- or carbohydrate-derived carbonyl compounds	

#### 3.1.1 ROS and DNA Damage

ROS cause a variety of DNA lesions and the resulting cellular responses involve complex crosstalk between multiple repairs and signaling pathways (Kay et al., 2019). ROS induce DNA damage such as the formation of 8-oxoguanine (Kay et al., 2019; Schaich and Van Houten, 2021), the most abundant oxidative DNA lesion, and other types of nucleotide oxidation, deamination, and lipid peroxidation-derived adducts. DNA repair pathways for oxidative DNA lesions include base excision repair and to a lesser extent, nucleotide excision repair (Freudenthal et al., 2017). Failure to repair oxidative lesions prior to replication can lead to mutations (Sasaki et al., 2020). Alternatively, ROS can produce single-strand DNA breaks directly or indirectly (through repair intermediates), replication stress, and the formation of double-strand breaks (DSBs) (Kay et al., 2019). DSB repair occurs by the error-prone non-homologous end-joining pathway or by homologous recombination repair (Kay et al., 2019). Upon the occurrence of DSBs induced by ROS, histone H2AX nearby DSBs is phosphorylated, and phosphorylated H2AX (*γ*-H2AX) induces DNA damage responses (Ishida et al., 2014). Polyphenols and flavonoids protect DNA from ROS-induced oxidative damage (Khalil Alyahya et al., 2021). DNA damage response networks including pathways leading to the formation of mutations and chromosomal aberrations are involved in cancer (Tanabe et al., 2021).

#### 3.1.2 ROS and Cancer

Cancer is the first or second leading cause of death before the age of 70 years in 112 of 183 countries, according to estimates from the World Health Organization (WHO) in 2019 (Organization, 2020; Sung et al., 2021). While ROS can be a cause of cancer, they can also have a protective effect: e.g., promotion of oxidative stress-induced cancer cell death caused by excessive ROS-induced oxidative damage, and tumor formation in redox-dependent and pro-oncogenic signaling pathways (De Logu et al., 2021; He et al., 2021). Many mechanisms are conserved in different types of cancer and have similar carcinogenic effects (Kay et al., 2019). ROS-induced DNA damage promotes inflammation and cancer (Helm and Rudel, 2020). Epithelial-mesenchymal transition (EMT), a transition of cells from epithelial to mesenchymal state, is the main hallmark of cancer malignancy (Hanahan and Weinberg, 2011; Tanabe et al., 2020; Tanabe et al., 2021). ROS production is involved in the EMT process in cancer (Kozak et al., 2020). Sustained chronic ROS is linked to the poor prognosis of cancer. The expression of NOX4, one of the main sources of ROS, is correlated with tumor size, lymphatic metastasis, and vascular invasion, and thus poor prognosis in gastric cancer (Du et al., 2019). Conversion of fibroblast growth factor receptor 2b (FGFR2b) to FGFR2c induces EMT and represses nuclear factor-erythroid 2 (NFE2) like basic leucine zipper (bZIP) transcription factor 2 (NRF2)-mediated detoxification of ROS, which leads to the progression of cancer (Katoh and Katoh, 2009). ROS mediate the transformation of the tumor microenvironment in radiotherapy-resistant gastric cancer (Gu et al., 2018). Chloroquine-induced ROS production inhibits autophagy and promotes EMT and malignancy in estrogen receptor-positive breast cancer cells (Rojas-Sanchez et al., 2021). Since autophagy inhibition could be a potential cancer treatment, cancer subtypes need to be considered in the application of therapeutics (Rojas-Sanchez et al., 2021). The evidence supports the involvement of ROS in treatment-resistant cancer. Some controversy regarding the role of ROS in cancer progression is ongoing; for example, heat shock protein 27 (HSP27), a stress-induced molecular chaperone, inhibits ROS accumulation, while its expression is associated with metastasis and poor prognosis in cancer (Nagata et al., 2013).

#### 3.1.3 ROS and Infection

Similar to the double role in cancer, the roles of ROS in infection represent the “double-edged sword” concept. The relationships between oxidative stress responses and coagulation in terms of SARS-CoV-2 infection are yet to be determined (Cecchini and Cecchini, 2020). Mesenchymal stem cells (MSCs) are one of the candidate therapies for COVID-19 to reduce inflammation and promote lung regeneration in severe COVID-19 patients (Rocha et al., 2020). MSCs express SOD, which converts superoxide anion to H_2_O_2_ and free oxygen, preventing the destruction of surrounding tissue by ROS from neutrophils and M1 macrophages (Jiang et al., 2016; Rocha et al., 2020). AOP379 regarding coronavirus infection and ROS has been developed as an OECD project (https://aopwiki.org/aops/379), in collaboration with an international consortium, the Modelling the Pathogenesis of COVID-19 Using the AOP Framework (CIAO) (Clerbaux, 2022). It is expected that research in this field will develop in the future, including involvement in the immune response of RNA signal networks.

### 3.2 ROS and Nanoparticles (NPs)

The AOPs leading to effects on the liver by titanium dioxide (TiO_2_) have been developed, where ROS generation is a KE in the AOP network (Brand et al., 2020). Exposure to TiO_2_ seems to trigger ROS generation and oxidative stress (Brand et al., 2020). The estimated cumulative dose of 10^2^–10^4^ mg/kg bw, or less than 10^3^ mg/kg bw of TiO_2_ induces ROS generation, or oxidative stress, respectively. In contrast, a wide range of the cumulative concentration (10^2^–10^12^ mg/kg bw) of TiO_2_ induces preneoplastic lesions (Braakhuis et al., 2021). Prolonged inhalation of TiO_2_ causes its deposition in the lung, which leads to ROS generation (considered as a KE) and oxidative stress (considered as another KE) in lung adenomas/carcinomas (Braakhuis et al., 2021). While nanomaterials potentially induce ROS (Jacobsen et al., 2008) and oxidative stress (Halappanavar et al., 2021), nanoparticle (NP)-based ROS-scavenging approaches have also emerged as nanomedicines for anti-inflammatory treatments (Huang et al., 2021). These ROS-scavenging NPs include catalytic NPs that have SOD-, CAT-, POD-, and glutathione-like enzyme activities, free-radical trapper NPs such as fullerene that captures ROS *via* conjugated double bonds, 2, 2, 6, 6-tetramethylpiperidinenoxyl (TEMPO) that captures ROS *via* the single electron on nitroxide, and redox ROS-scavenging NPs such as curcumin or bilirubin NPs (Nash and Ahmed, 2015; Huang et al., 2021). Specific ROS-targeting NPs as drug delivery systems, where solubility changes by ROS or extended-release are utilized for anti-cancer therapy, have been developed as well (Kim et al., 2015). Conversely, carbon black NPs have been shown to induce ROS, oxidative DNA damage, and a ROS-specific mutation spectrum (Jacobsen et al., 2007; Jacobsen et al., 2008; Jacobsen et al., 2011; Modrzynska et al., 2018). In addition, a SOP for assessment of ROS generation using DCFH-DA acellular assay was developed (Boyles et al., 2022). Future studies with stable detection of ROS induced by nanoparticles would reveal the controversial effects of NPs in terms of the relationship between ROS and pathological changes.

### 3.3 ROS and Radiation Toxicity

Low level radiation is omnipresent due to exposure to natural radionuclides, cosmic and solar radiation and widespread use in various industrial applications (Zdrojewicz et al., 2016). It comes in different forms including both ionizing (e.g., alpha particles, heavy ions, neutrons, beta electrons, gamma photons, X-rays and UVC) and non-ionizing radiation (e.g., UVB, UVA, visible light, microwave, radio and other low-frequency radiation). Ionizing radiation arises from many sources including natural (e.g., Naturally Occurring Radioactive Materials (NORM) and cosmic rays), medical (e.g., radiotherapy), diagnostic (e.g., computed tomography) and anthropogenic activities such as mining and milling (e.g., Technologically Enhanced Naturally Occurring Radioactive Material (TENORM)). Severe adverse effects of ionizing radiation has predominantly been associated with acute exposures to high radiation dose rates from nuclear accidents and nuclear detonations, albeit prolonged exposure to low radiation dose rate is considered relevant for a number of chronic pathologies. Non-ionizing radiation such as solar UV (UVA and UVB) and anthropogenic use of high-energy UVC radiation in disinfection etc. are potential sources for both acute and chronic effects. Although UV radiation is considered highly relevant for human and environmental health, ionizing radiation are often highlighted for its high ROS-inducing potential.

When ionizing radiation interacts with the high-water content of cells, the ionization events generate a variety of molecular species from water radiolysis which include the hydrated electron, radicals such as hydroxyl, hydride, superoxide, and molecular species such as H_2_O_2_ and di-hydrogen (Gebicki, 2016). The production of ROS from these interactions is therefore secondary to the initial physical interaction with biological matter (Alizadeh et al., 2015). Once formed, these species can also induce damage, alter the redox environment in proximity of the track and/or mimic signaling pathways that involve ROS produced by other mechanisms such as mitochondrial sources (Breitenbach and Eckl, 2015; Sies, 2015; Tharmalingam et al., 2017). The extent of ionization events and follow-on ROS-induced toxicity pathway activation can be influenced by the particle size and initial kinetic energy which is often a function of dose of exposure and dose-rate of delivery. In the case of photons that have no mass, the incident particle is an electron with an energy related to the incident photon wavelength. The physiological consequences are wide-ranging depending on the extent and specific site of damage. Studies have shown oxidative stress from radiation exposure can lead to neurotoxicity closely associated with Alzheimer’s disease, developmental disorders (Cheignon et al., 2018), reproductive disruption (Xie et al., 2019; Song et al., 2020), growth inhibition (Xie et al., 2019) and cardiovascular diseases (Ahotupa, 2017). However, studies have also shown that ROS production is not necessarily detrimental and can have an adaptive role, particularly at low levels, enhancing the resistance of cells to oxidative damage from higher levels of radiation exposure (Buonanno et al., 2011). Additionally, co-exposure to radiation with other environmental stressors and cellular toxins often causes augmented biological effects mediated through ROS production (Salbu et al., 2019). Additionally, factors such as endogenous production of ROS, nutritional and antioxidant status, age, life stage differences and individual as well as epigenetic variations are known modulators of ROS formation and further influence radiation-induced toxicity.

### 3.4 Biomarkers of ROS

Biomarkers have been defined by the U.S. Food and Drug Administration and the National Institutes of Health as measurable characteristics, which are evaluated as indicators of a biological process, pathogenic process, or pharmacological response (Califf, 2018). Biomarkers of effect are crucial for determining the formation of ROS in the AOP and their role in the AOP as they can often serve as a readout for KEs or associated events in the causal chain of events leading to an AO. Macromolecular degradation products might serve as biomarkers of the effect of ROS in a causal chain of events leading to the AO such as cellular/organ structural degeneration. In the case of gene expression alterations, it may be more suitable to call the event an associated event rather than a KE, since the genes induced may encode cytoprotective proteins that seek to mitigate it. Thereby differences in organ-specific antioxidative defenses and basic ROS levels need to be considered (Scandalios, 2005). Several biomarkers of ROS can be assessed in dependence on *in vitro* or *in vivo* contexts, and at different levels of biological organization (Ho et al., 2013). The products of *in vivo* ROS can be measured in body fluids such as blood or urine. Examples of such are biomarkers concerning lipid peroxidation like MDA, protein damage like 3-nitro-tyrosine (3-NO_2_Tyr), DNA/RNA damage like 8-OHdG or 8-nitro-guanine (8-NO_2_Gua) or general biomarkers such as glutathione (oxidized/reduced ratio) (Porter et al., 1995; Meagher and FitzGerald, 2000; Smith et al., 2011; Steffensen et al., 2020). F_2_-isoprostanes, prostaglandin-like compounds formed by non-enzymatic free radical-induced peroxidation of arachidonic acid, can be biomarkers for monitoring ROS and oxidative stress (Milne et al., 2011; Ma et al., 2017). In addition, *in vitro* ROS can directly be measured by using fluorescent dyes. Here, fluorescent probes such as DCFH-DA detected cytosolic ROS (Xie et al., 2019; Song et al., 2020), while dihydrorhodamine 123 (DHR123) and C11-BODIPY were applied for the detection of mitochondrial and lipid peroxidation-related ROS, respectively (Gomes et al., 2017; Song et al., 2020). Also, changes in antioxidative gene expression can serve as the first indication of exposure exceeding basal defense levels. Differential expression of genes coding for antioxidative enzymes such as *SOD1*, heme oxygenase 1 (*HMOX1*), oxidative stress-induced growth inhibitor 1 (*OSGIN1*), *NQO1* or glutamate-cysteine ligase modifier (*GCLM*) can be indicative, although there is no established signature of differential gene expression profile for ROS (Yang and Chitambar, 2008; Gornicka et al., 2011; Yi et al., 2014; Tsai et al., 2017). There is not a consistent understanding of how changes in expression of genes indicate ROS levels. For instance, *NQO1* is up-regulated while *CAT* is down-regulated in nonalcoholic steatohepatitis livers (Gornicka et al., 2011). *HMOX1* is up-regulated and *CAT* is down-regulated by gallium nitrate in human lymphoma cells (Yang and Chitambar, 2008). Moreover, levels of antioxidant enzymes [SOD, CAT, POD, GST, GPX or glutathione-reductase (GR)] can be determined as indicators of redox state and oxidative stress (Scandalios, 2005). Especially, gene expression changes might be used as a first-line screening for a MoA of *in vitro* ROS. This was recently exemplified in a study examining the MoA of herbal medicines. Here, ROS-dependent induction of antioxidative gene expression serves as an event in the generated putative AOP “Disturbance of oligodendrocyte differentiation/maturation leading to intellectual disability due to alterations in white matter” (Klose et al., 2022). For the determination of the role of ROS as a KE within an AOP, the most sensitive biomarkers of ROS need to be utilized. Either a direct measurement of ROS or the use of some of the most sensitive gene expression biomarkers such as *HMOX1* and *NQO1* would appear to be the optimal methods. These must be measured in a time-based manner relative to the AO to carefully distinguish whether the ROS formation is a key or associated event in relation to the AO. Furthermore, care must be taken with the biological system, and in particular *in vitro* where the high partial pressure of oxygen can result in the formation of ROS which may not be relevant in the low oxygen partial pressure environment of the organ (Ortiz-Prado et al., 2019).

## 4 Creation of Harmonized ROS-Related KEs

**FIGURE 1:**
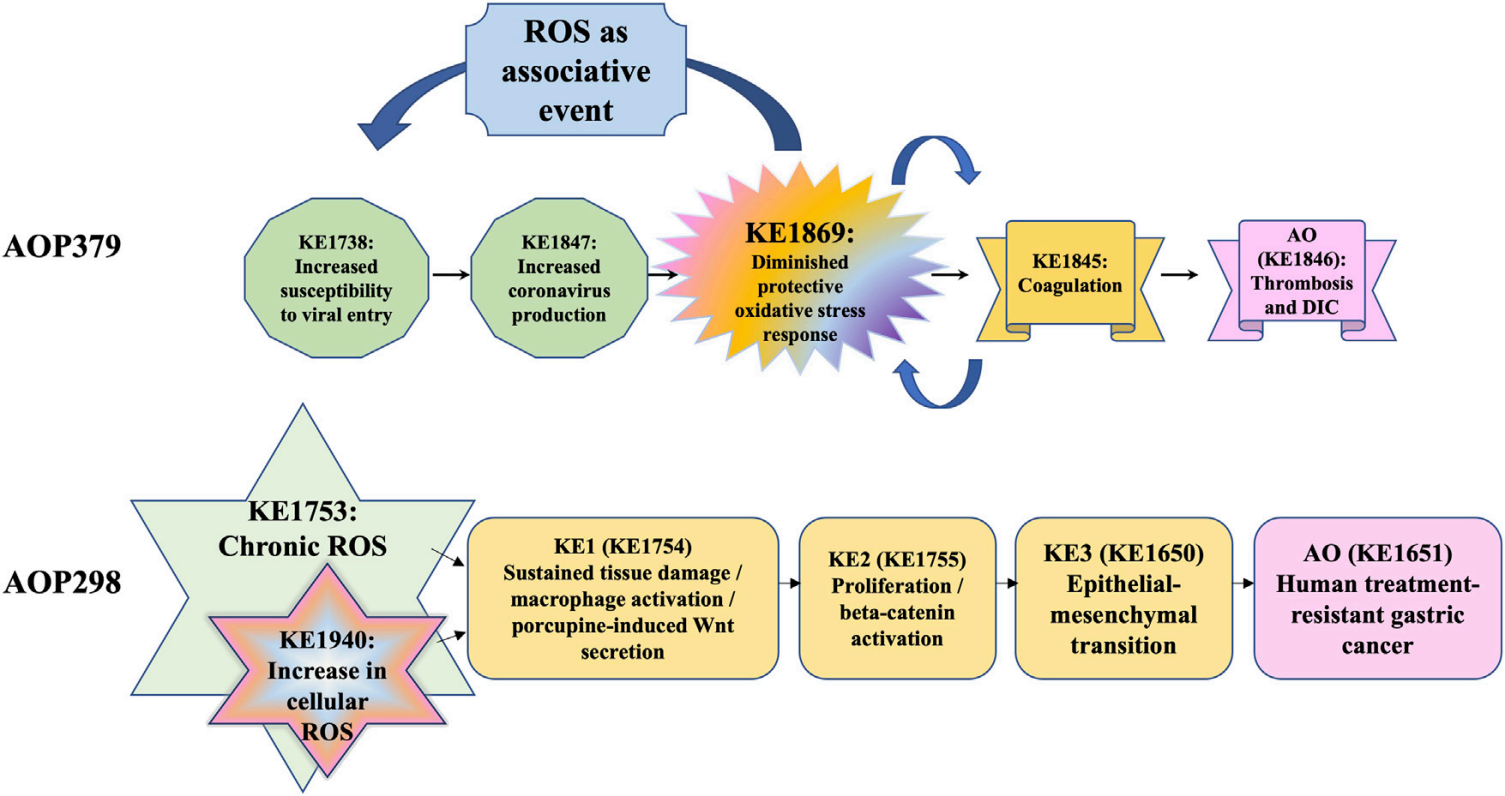
Examples for the concept of the ROS as a KE and associative event in AOPs *DIC: disseminated intravascular coagulation.

The Mystery of ROS consortium aims to create harmonized ROS-related KEs. One umbrella event capturing all components such as “imbalance of oxidative stress processes” could be considered. Alternatively, ROS and RNS could be combined to “reactive oxygen and nitrogen species (RONS)” and ADR represented separately, as the role of ADR in disease/adversity progression is well-defined. There is an obvious challenge of providing sufficient specificity in the KE description to balance producing a general KE that can also be reused in the AOP framework as a modular unit in different related pathways. Similarly, another challenge is identifying which measurement methods to include in the description such that the KE remains broad and not overly specific. Directionality of KEs defined as either “up” or “down” would also need consideration. It has been considered whether the terminology of “up-regulation of ROS” and “increase in ROS” is different or not, since “up-regulation of ROS” rather means active production of ROS in cellular mechanism, while “increase in ROS” captures both aspects of the excessive production of ROS and decrease in ADR. Active regulation of ROS, which the term “up-regulation of ROS” may imply, may fit better to certain cellular or molecular reactions in disease progression (e.g., inflammatory responses, immune responses, etc.), however, may not always fit in the events of ROS produced or formed directly due to the oxidative reactions involving exogenous stressors. Naming such as “excessive RONS production” would be another possibility, however, would need a clearer definition of what “excessive” means and is highly dependent on the ADR status, and therefore challenging to determine without taking into account downstream events of the AOP to define a threshold for what is considered “excessive”. The consortium also discussed “Depletion of protective oxidative stress response,” this term was not favored as it indicates that the protective responses can be exclusively associated with one mechanism of action. The ADR represents both depletion of cellular antioxidants and induction of protective enzymes that regenerate cellular reduction potential (e.g., reducing oxidated proteins) or enzymatically reduce a radical species. The term “depletion” therefore does not cover the diversity of the ADR system, and terminology such as “exhaustion” or similar terminology such as “diminished” may be more appropriate. An alternative naming considered would be “insufficient antioxidant defenses” which is defined in terms of a state or condition that may not be easily determined. However, none of these terms cohere well with defining the directionality for easy integration into the AOP-Wiki (https://aopwiki.org). As both depend on the RONS production (endogenous/exogenous) or the demonstration of the onset/triggering of downstream KEs to indicate a departure from the condition “sufficient”, they may thus not be ideal terms for the complex or totality of the ADR. A general term such as “increase, (protective) oxidative defense response” (directional) or “altered (protective) oxidative defense response” (undirectional) would capture the naming of events crucial for the AOP itself (i.e., a KE) as well as modifying factors that would not necessarily be considered as a KE in the AOP. The main objectives of KE harmonization described in this manuscript were to estimate existing knowledge of general ROS regarding the AOP framework and its application and gather ideas and input from the broader scientific community that can help guide and direct further development of the AOP framework. These initiatives of the workshop have provoked a discussion among research scientists in moving the Science of AOPs forward to consider how best to use and optimize the ROS-driven oxidative stress in the regulatory AOP framework. Therefore, it is relevant to hazard and risk assessment that research and regulatory communities identify current limitations of undefined ROS KEs and their qualitative and quantitative implications to guide data curation to ensure consistency for oxidative stress-specific AOPs and their applications. Identification of the therapeutic targets or prediction of adverse effects of therapeutics in diseases utilizing the AOP framework would be one of the future directions (Figure 1).

## 5 Conclusion

ROS produced in response to stimuli play various roles and have many faces in human health and diseases. The beneficial roles and adverse effects of ROS should be considered to solve the puzzles of AOP constructs with existing insights in the ROS field. The mystery of ROS consortium continues the effort to harmonize the events in ROS-related networks. The aim of this review was to clearly show where ROS are likely to be a KE within an AOP, and the role of the ADR to mitigate the effect of ROS and thus modulate the magnitude of the associated AO. There is a clear need to differentiate in an AOP where ROS are a KE and where they are associated events. This can be done by understanding the temporal sequence of events associated with ROS and where they occur relative to the AO and the KEs likely associated with the MIE (Figure 1). Further discussion would continue in the Mystery of ROS consortium in the future.
